# Familiarity and Voice Representation: From Acoustic-Based Representation to Voice Averages

**DOI:** 10.3389/fpsyg.2017.01180

**Published:** 2017-07-14

**Authors:** Maureen Fontaine, Scott A. Love, Marianne Latinus

**Affiliations:** UMR7289, Centre National de la Recherche Scientifique, Institut de Neuroscience de la Timone, Aix-Marseille Université Marseille, France

**Keywords:** familiarity, voice, identity, average, speech, recognition, prototypes, vowels

## Abstract

The ability to recognize an individual from their voice is a widespread ability with a long evolutionary history. Yet, the perceptual representation of familiar voices is ill-defined. In two experiments, we explored the neuropsychological processes involved in the perception of voice identity. We specifically explored the hypothesis that familiar voices (trained-to-familiar (Experiment 1), and famous voices (Experiment 2)) are represented as a whole complex pattern, well approximated by the average of multiple utterances produced by a single speaker. In experiment 1, participants learned three voices over several sessions, and performed a three-alternative forced-choice identification task on original voice samples and several “speaker averages,” created by morphing across varying numbers of different vowels (e.g., [a] and [i]) produced by the same speaker. In experiment 2, the same participants performed the same task on voice samples produced by familiar speakers. The two experiments showed that for famous voices, but not for trained-to-familiar voices, identification performance increased and response times decreased as a function of the number of utterances in the averages. This study sheds light on the perceptual representation of familiar voices, and demonstrates the power of average in recognizing familiar voices. The speaker average captures the unique characteristics of a speaker, and thus retains the information essential for recognition; it acts as a prototype of the speaker.

## Introduction

Voice production involves the whole body, and as such the voice captures the physical characteristics of a speaker, making it uniquely individual (Kreiman and Sidtis, 2011). Voices are primary social signals used for the recognition of familiar individuals—a key aspect of social interaction. Recognition of familiar voices, which transmit crucial information about a person, is a widespread ability that has evolved in response to different environmental and behavioral demands (Sidtis and Kreiman, 2012). Over a lifetime, human listeners encode hundreds of voices, and maintain them in long-term memory. Despite being of interest to a large variety of disciplines, from psychology to forensic sciences through technology, the bases of this remarkable ability are not fully understood (Kreiman and Sidtis, 2011; Podesva and Callier, 2015). We report two experiments in which we investigated the neuropsychological processes involved in voice identity recognition.

Voice identity perception refers to both the recognition of unfamiliar and familiar voices (Van Lancker and Kreiman, 1987; Van Lancker et al., 1989). While familiar voice recognition refers to the act of identifying a known voice, unfamiliar voice recognition refers either to the act of deciding whether a previously heard but unknown voice is presented again or the act of discriminating between two voices presented successively. Familiar voice recognition encompasses several levels of familiarity (Kreiman and Sidtis, 2011; Blank et al., 2014) among them personally-familiar voices and famous voices are those voices a listeners has known for a long period of time, heard in different contexts and associated with multiple pieces of semantic information and feelings. To overcome difficulties in using personally-familiar or famous voices, researchers often rely on the use of voices learned during the course of an experiment (e.g., Von Kriegstein and Giraud, 2006; Winters et al., 2008; Andics et al., 2010; Latinus and Belin, 2011; Latinus et al., 2011). In general, voice learning paradigms involve participants learning to associate names or corresponding faces to the vocal material produced by different speakers (Von Kriegstein and Giraud, 2006; Winters et al., 2008; Andics et al., 2010; Latinus and Belin, 2011; Latinus et al., 2011). Voice learning durations vary greatly across studies: from a single training session lasting about 30 min (Von Kriegstein and Giraud, 2006), to multiple training sessions spanning several days (Winters et al., 2008; Andics et al., 2010; Latinus and Belin, 2011; Latinus et al., 2011). Training sessions often include several steps: (1) a familiarization step in which participants learn the association between the voice and name/face; (2), a recognition task in which participants perform an identification task; (3) an evaluation phase, identical to the recognition phase except that participants do not receive feedback. Training stops either after participants reach a predefined number of training sessions, or after they reach a pre-defined criterion in their performance. Thus, laboratory learned voices, also referred to as “trained-to-familiar voices” (Kreiman and Sidtis, 2011), form a third level of familiarity, for which familiarity is acquired from a learning phase, over a relatively short amount of time (days/weeks) and little semantic information. Familiar voice recognition and unfamiliar voice discrimination are separate abilities that can be selectively impaired (Van Lancker and Kreiman, 1987; Van Lancker et al., 1989). At the time, Van Lancker and colleagues argued that familiar voice recognition was impaired by lesions to the right parietal cortex, while a deficit in unfamiliar voice discrimination was associated with lesion of the temporal lobe of either hemisphere. Familiar voice recognition, despite becoming increasingly more efficient during development (Spence et al., 2002), is present at birth (Decasper and Fifer, 1980) while voice discrimination is only mature at the age of 10 (Mann et al., 1979). Therefore, multiple studies agree that the perception of familiar and unfamiliar voices is the result of different mechanisms. Yet, whether laboratory training is rich enough to create artificially-learned voices that match everyday learning remains an open question. A partial answer comes from a recent review of neuroimaging studies in healthy adults, which highlights different neural substrates involved in the recognition of trained-to-familiar voices and familiar person recognition (including both personally-familiar and famous individuals; Blank et al., 2014). Familiar person recognition involved an extended brain network in the bilateral anterior temporal lobe and right posterior cingulate cortex; on the contrary trained-to-familiar voices activated bilateral frontal, temporal and parietal regions. Thus, it appears that trained-to-familiar voices do not behave as truly familiar voices. The studies presented here aim at strengthening our understanding of voice perception by looking at how voices are encoded in the brain, and how different types of familiarity influence this. In two experiments we investigated the perceptual representation of voices of varying degrees of familiarity.

Despite high intraspeaker variability, listeners are relatively good at perceiving voice identity even across large variations in sound quality and content (Van Lancker et al., 1985; Schweinberger et al., 1997a; Nakamura et al., 2001; Belin et al., 2011; Kreiman and Sidtis, 2011). The combination of acoustic cues carrying information about a speaker's identity is complex and largely unknown. Most research assumed and sought to identify a fixed set of acoustic cues implicated in voice recognition (e.g., Murry and Singh, 1980; Lavner et al., 2000; Schweinberger, 2001; reviewed in Kreiman and Sidtis, 2011). For instance, it has been proposed that familiar speaker identity is mainly conveyed by three acoustic features: the fundamental frequency and the third and fourth formants (Lavner et al., 2001). More recently, a study using professional impersonators demonstrated that voice identity was mainly associated with acoustic features reflecting the anatomy of the vocal tract, such as the difference between the fourth and fifth formants (Lopez et al., 2013). Using trained-to-familiar voices, adaptation to identity was found only when the original configuration of the voice was preserved, suggesting that voice identity is represented in a complex pattern encompassing multiple acoustic information (Latinus and Belin, 2012). To date, no studies have been able to describe a universal set of acoustical parameters that could reliably allow the identification of a human voice, suggesting that familiar voices are rather encoded as a “Gestalt-like” complex pattern (Kuwabara and Sagisak, 1995; Belin et al., 2011; Kreiman and Sidtis, 2011; Sidtis and Kreiman, 2012; Schweinberger et al., 2014). Consistently, identity priming in which the probe and test stimuli differed physically only facilitates familiar voice recognition, suggesting that familiar, but not unfamiliar, voices are represented as a complex abstract pattern independent of phonetic and linguistic information (Schweinberger et al., 1997b). Yet, the nature of the processes involved in representing familiar voices remains unknown. Here, we asked whether this unique voice pattern could be approximated by the average of varied utterances produced by a single speaker. This question was addressed for both famous and trained-to-familiar voices.

We explored the processes involved in familiar and trained-to-familiar voice recognition from very short voice samples. We predicted that listeners would recognize familiar voices in reference to a unique vocal pattern, well approximated by the average of multiple utterances produced by a single speaker, e.g., the “speaker average.” This speaker average combines all characteristics of a speaker's voice and represents the unique vocal pattern of familiar speakers. Based on prior work on face perception (Burton et al., 2005; Jenkins and Burton, 2011), we proposed that averaging several utterances from the same speaker could approximate the speaker's vocal pattern by preserving the idiosyncratic information about the speaker, while eliminating environmental and intraspeaker variability. Under this assumption, identity recognition performance is expected to be better for speaker averages created artificially than for original voice samples. Because there are differences in the neural networks involved in processing trained-to-familiar and familiar voices (e.g., Blank et al., 2014), this hypothesis was tested for both learned (experiment 1) and famous (experiment 2) voices.

Here we investigated the neuropsychological processes involved in voice identity perception. In two experiments, we tested the hypothesis that familiar voice recognition involves speaker averages. Participants performed three-alternative forced-choice (3AFC) identification tasks on short voice samples including original vowels uttered by the familiar speakers and the speaker averages created through morphing several different vowels uttered by the same speaker. In experiment 1, the voice samples were from three trained-to-familiar female speakers. In experiment 2, the voices were from three famous French public personalities. We predicted that identity recognition on the speaker average would be facilitated with respect to the original voice samples (accuracy increases and/or response times decrease). To this aim, we manipulated the number of utterances included in the speaker averages by averaging together different numbers of vowels uttered by a single speaker, expecting a linear relationship between behavior (reaction times and accuracy) and level of averageness (number of different vowels averaged). All the studies described in the current manuscript have been carried out in accordance with The Code of Ethics of the World Medical Association (Declaration of Helsinski) and were approved by the local ethics committee (Comité de Protection des Personnes Sud Méditerranée I).

## Experiment 1: individual averages in trained-to-familiar voice recognition

### Methods

#### Participants

Thirteen native French speakers (mean age ± standard error of mean (s.e.m.): 21.9 ± 1.17; 5 males) with self-reported normal audition, normal vision and without cognitive disorders, participated in the experiment. They provided written informed consent and received monetary compensation for their participation.

#### Stimuli

Voice samples were drawn from a database of native French-Canadian voices (Baumann and Belin, 2010). Original recordings were made in the multi-channel recording studio of Secteur ElectroAcoustique in the Faculté de musique, Université de Montréal. The speakers were instructed to utter multiple vocalizations from sentences to vowels, through nonverbal vocalizations. For the purpose of the experiment, we extracted French vowels (IPA: [a], [e], [i], [o], [y], [u]) produced in isolation manually with Audacity (Copyright (C) 1989, 1991 Free Software Foundation, Inc.) from the original recordings of three female voices. For the three to-be-learned voices, we extracted from the original recordings 6 stimuli (one version of each of the 6 vowels, used in the experiment) as well as stories (*n* = 2) and words produced in isolation (*n* = 23); the same words and stories were used in the training phase for all trained-to-familiar voices. The first story comprised 43 words and lasted on average 16.77 s (±0.35 s); the second story comprised 59 words and lasted on average 20.704 s (±0.25 s). All stimuli were normalized in energy with Matlab-R2013 (The MathWorks, Inc., Natick, MA, USA). Vowel duration for the three to-be-learned voices was 464 ms ± (SD) 31 ms. Average duration of vowel [a] across the 10 speakers was 427 ms ± (SD) 108 ms.

##### Speaker averages

For each of the three speakers, five speaker averages, with varying level of averageness, were created by morphing 2, 3, 4, 5, or 6 different vowels ([a], [e], [i], [o], [y], [u]) produced by a single speaker using STRAIGHT (Speech Transformation and Representation by Adaptative Interpolation of weiGHTed spectrogram; Kawahara and Matsui, 2003) operating in the Matlab2013 environment (MATHWORKS Inc., Natick, MA). STRAIGHT performs an instantaneous pitch-adaptive spectral smoothing in each stimulus to separate the contributions of the glottal source and the supralaryngeal filtering to the voice signal. Voice stimuli are decomposed by STRAIGHT into five parameters that can be manipulated independently: f0, i.e., the perceived pitch, formant frequency, duration (ms), spectrotemporal density and aperiodicity. Landmarks to be put in correspondence across the different vowels were manually identified in the time-frequency space. Temporal anchors were the beginning and end of production. Frequency anchors were the first, second, third and fourth formants at onset and offset of phonation; Praat (http://www.praat.org/) was used to ease the identification of the first four formants (Boersma and Weenink, 2017). Speaker averages were then generated based on the interpolation (linear for time and aperiodicity, and logarithmic for the other parameters) of these time-frequency landmarks. For each vowel included in the average, all parameters extracted by STRAIGHT, but duration, were given equal weight e.g., a weight of 1/2 on vowels [a] and [u] for speaker 1 was used to generate the speaker average with an averageness level of 2; following the same logic, a weight of 1/3, 1/4, 1/5, and 1/6 was used to create speaker average of 3, 4, 5, and 6 vowels, respectively). The same weight of 1/60 was applied to the duration parameter, resulting in all speaker averages having the same duration of 471 ms. For each level of averageness, only one combination of vowels was used and this was held constant across speakers, e.g., the individual average of 2 vowels was always created by morphing [a] and [u].

Acoustic measurements (fundamental frequency, formant dispersion and HNR) for the learned and famous voices are reported in Table 1.

**Table 1:** Acoustic measurements for trained-to-familiar voices and famous voices.

	**Trained-to-familiar voices**				**Famous voices**		
	**F0 (Hz)**	**FD (Hz)**	**HNR (dB)**		**F0 (Hz)**	**FD (Hz)**	**HNR (dB)**
**VOICE 1**				**VOICE 1**			
Original Vowel	220	1,132	17	Original Vowel	141	1,048	15
Morphs - 2	222	1,107	21	Morphs - 2	108	1,026	17
Morphs - 3	225	1,024	20	Morphs - 3	126	1,039	16
Morphs - 4	223	1,092	21	Morphs - 4	122	1,002	16
Morphs - 5	223	1,139	22	Morphs - 5	119	1,023	18
Morphs - 6	223	1,107	22				
**VOICE 2**				**VOICE 2**			
Original Vowel	243	1,088	27	Original Vowel	141	1,048	15
Morphs - 2	251	1,216	36	Morphs - 2	108	1,026	17
Morphs - 3	233	948	26	Morphs - 3	126	1,039	16
Morphs - 4	243	1,116	34	Morphs - 4	122	1,002	16
Morphs - 5	242	1,156	33	Morphs - 5	119	1,023	18
Morphs - 6	242	1,135	34				
**VOICE 3**				**VOICE 3**			
Original Vowel	214	1,089	14	Original Vowel	144	1,067	18
Morphs - 2	223	1,126	19	Morphs - 2	114	1,080	20
Morphs - 3	207	1,094	19	Morphs - 3	146	1,028	17
Morphs - 4	214	1,110	20	Morphs - 4	138	1,072	20
Morphs - 5	216	1,126	20	Morphs - 5	132	1,053	20
Morphs - 6	214	1,113	20				

*Note that famous voices were that of male speakers and trained-to-familiar voices were from female speakers*.

#### Procedure

Subjects sat in front of a screen in a quiet room. The experiment was presented using Psychtoolbox in the Matlab 2007 environment. Stimuli were delivered binaurally through Beyerdynamics (DT770 pro, beyerdynamic GmbH & Co. KG, Heilbronn, Germany) headphones with a Steinberg UR22 (Steinberg Media Technologies GmbH, Hamburg, Germany) soundcard.

##### Voice learning

Participants initially learned to recognize the same three female voices (chosen at random from the 10 female voices) using a standard three-step learning procedure, repeated over several days until a predefined criterion (global performance above 66%—corresponding to the discrimination threshold in a 3-AFC task; Kingdom and Prins, 2010) was reached (Latinus et al., 2011). The three-step training session lasted about 20 min. In an attempt to produce a relatively ecological learning of vocal identity, participants heard different vocal items produced by the three speakers, including sentences, words and vowels. The three-steps were as follows: (1) a familiarization phase during which subjects were instructed to listen carefully to stories voiced by each speaker and learned to associate a name presented on the screen with a particular voice; (2) a learning phase, during which, subjects performed a 3-AFC identification task on both words (*n* = 23) and vowels (*n* = 6), presented once—visual feedback was provided on their response. After an incorrect response the sound was repeated and the correct answer given; (3) a test phase in which only vowels were presented and subjects performed the 3-AFC without feedback; each token was repeated 9 times. In phase 2 and 3, stimuli were presented in random order with a stimulus onset asynchrony (SOA) varying between 2.1 and 2.5 s. Participants were instructed to respond as fast and accurately as possible. On average, training lasted 5.8 days (range 3–10). Performance at the last session (mean ± s.e.m. [bootstrapped 95% confidence interval - 95%CI] = 71% ± 1.17 [69% 73%]) was well above chance level [33%; *T*_(12)_ = 32.46, *p* < 0.001], confirming that participant learned to recognize the three female voices.

##### Identification task

After voice learning, participants took part in a 3-AFC identification task on vowels and speaker averages (averageness levels 2, 3, 4, 5, and 6). Stimuli were repeated in order to keep the number of trials per condition constant. Original vowels (*N* = 6) were repeated 3 times, leading to 18 trials for the vowel condition. For each level of averageness (2, 3, 4, 5, or 6), we generated one speaker average, which was then repeated 18 times. There were a total of 324 trials (6 vowels repeated 3 times ^*^ 3 voices, 5 averages repeated 18 times ^*^ 3 voices) presented randomly to the participants; ISI varied randomly between 2 and 2.3 s. Participants were asked to press one of three keyboard keys corresponding to the learned identities. The response keys were the same as those used in the training procedure and corresponded to the first letter of the learned names. They were instructed to respond as fast and accurately as possible; decision did not interrupt the presentation of the voice. The experiment took approximately 20 min including short breaks every 5 min.

Accuracy and response times, recorded from voice onset, were collected for each participant.

#### Statistical analysis

In order to confirm that participants did indeed recognize the voices, we assessed participants' accuracy against chance level (33%); to do so one sample *t*-tests were run for each of the 6 average levels (Table 2).

**Table 2:** Recognition of trained-to-familiar and familiar voices.

	**Vowels per prototype**					
	1	2	3	4	5	6
**TRAINED-TO FAMILIAR VOICES**						
T (12)	10.19	2.88	4.85	3.83	4.58	2.97
*p*	<0.0001	0.014	0.004	0.002	0.0006	0.012
Cohen's dz	2.8266	0.7996	1.3440	1.0609	1.2691	0.8224
**FAMILIAR VOICES**						
T (12)	7.52	5.2	14.8	8.65	8.62	
*p*	<0.0001	0.0002	<0.0001	<0.0001	<0.0001	
Cohen's dz	2.0858	1.442	4.1036	2.3984	2.3916	

*One-sample t-test against chance level*.

In order to test the hypothesis of a linear relationship between performance and level of averageness (e.g., number of utterances per average), which would indicate that familiar voice recognition involved speaker averages, response times and performance were analyzed using a regression coefficient analysis (e.g., Pfister et al., 2013). A linear regression between level of averageness (predictor or independent variable) and the dependent variable (i.e., accuracy and RTs) was calculated for each participant. For each individual participant; we extracted the coefficients (slope and intercept) resulting from the linear regression. Note that for accuracy, a positive slope reflects that performance improved with level of averageness, that is voice morphs, with increasing number of vowels, were recognized more accurately than original voices; conversely, a negative slope indicates that voice morphs were recognized less accurately than original voices. For RTs, a positive slope indicates shorter RTs for the voice morphs than the original vowels.

Slopes of the regression analysis were then tested against a population mean of 0 (no slope) via one-sample *t*-tests (one for accuracy, one for RTs) to assess the significance of the linear regression at the group level. Statistical significance of the one sample *T*-test was assessed via bootstrapping because the regression coefficient distributions violated the normality assumption: participants were sampled with replacement, a *T*-value was estimated for the random sample and this operation was repeated 10,000 times. Threshold *T*-values were extracted by taking bootstrap values corresponding to the 95% confidence interval of the sorted bootstrapped T values (accuracy threshold [−3.07 1.82], RTs threshold [−2.66 1.92]). The test was considered significant if the real *T*-value fell outside the 95%CI obtained by bootstrapping. P values were calculated by counting the number of times the random samples gave value of T greater than the empirical one. Reported effect size is the Cohen's dz, e.g., the standardized mean difference effect size for within-subject designs as described in Lakens (2013).

### Results and discussion

Participants performed a 3-AFC identification task on vowels and speaker averages. Speaker averages were built by morphing together 2, 3 4, 5, or 6 different vowels produced by the same speaker. Speaker identification was significantly above chance level (33%) in all conditions [all *T*_(12)s_ > 2.88; all ps < 0.015–Table 2]. This result highlights the robustness of speaker recognition by demonstrating that listeners were able to identify speakers on brief vowels, and generalize their strategy to stimuli that have never been heard before, as is the case of the speaker averages.

**Figure 1:**
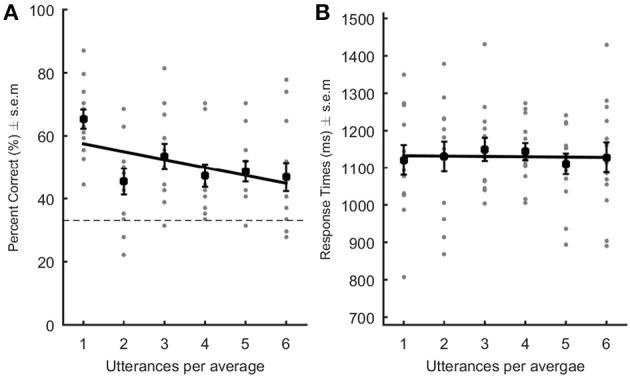
Performance in the recognition of trained-to-familiar voices. Percent correct **(A)** and response times (**B**; ms) are represented as a function of the level of averageness (i.e., number of utterances per voice average). Gray dots represent each participant's data point. The black square represents the average performance across listeners. In **(A)**, the dotted line indicates chance level. Black lines: linear regression built using the average slope and intercept values obtained after performing the linear regression in each subject. The slope was significantly decreasing in **(A)** indicating that performance worsened with increasing number of utterances per average.

Correct responses and reaction times were analyzed by means of a linear regression at the participant's level. A negative linear relationship occurred between the number of utterances per speaker average and percent of correct identification {average slope ± s.e.m. [95% CI] = −2.53 ± 0.44 [−3.32 −1.66]; *T*_(12)_ = −5.71; *p* = 0.0011; Cohen's dz = 1.58–Figure 1A}: accuracy decreased with an increasing number of vowels per average. Response times were not significantly correlated with number of utterances making up the speaker averages {average slope ± s.e.m. [95% CI] = −0.87 ± 8.35 [−15.86 15.704]; *T*_(12)_ = −0.104; *p* = 0.92; Cohen's dz = 0.03–Figure 1B}. These results show that, contrarily to our expectations, speaker averages were not better recognized than original vowels, at least for trained-to-familiar voices; they were actually more often wrongly identified.

As previous research has highlighted differences in the processing of familiar and trained-to-familiar voices, in the next experiment, we explored the same hypothesis with famous voices; that is we tested whether famous voices are represented globally as complex patterns, well approximated by the average of multiple utterances produced by a single speaker.

## Experiment 2: individual average in famous voice recognition

### Methods

#### Participants

The same 13 participants participated in the experiment.

#### Stimuli

Voice samples in experiment 2 were extracted from speeches found in video or radio sources from three famous male French people. Five different vowels ([a], [e], [ə], [o], [u]) were extracted from continuous speech downloaded from the Internet, and were normalized for energy. The five vowels were then morphed together to create speaker averages comprising 2, 3, 4, or 5 vowels following the procedure described in Section Speaker averages. Stimulus duration was 362 ms. For each level of averageness (2−5), only one combination of vowels was used and this was held constant across speakers.

#### Procedure and analysis

Procedure was similar to that of Experiment 1. Participants were exposed to the famous voices in order to freshen-up their memories as follows: (1) they listened to a short sentence from the 3 famous speakers, (2) they performed a 3AFC identification task on vocal samples (15 trials) that were not used in the actual experiment. Behavior was not recorded during training.

After this first exposure, participants took part in the 3AFC identification task on vowels and speaker averages comprising 2, 3, 4, or 5 different vowels. Original vowels were repeated 3 times, and each speaker average was presented 15 times, leading to a total of 225 stimuli (5 vowels ^*^ 3 times ^*^ 3 voices + 4 averages # 15 times ^*^ 3 voices). Stimuli were presented in a random order with an ISI varying randomly between 2 and 2.3 s. Participants were asked to press one of three keyboard keys different from those used for the learned identities; the key/identity association was presented during exposure. The experiment took approximately 15 min with a break every 5 min.

**Figure 2:**
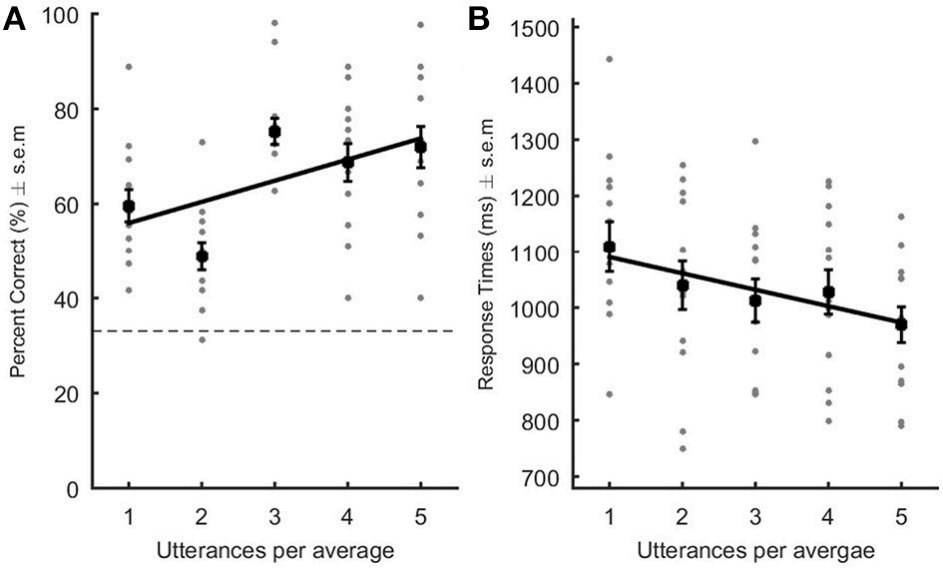
Performance in the recognition of famous voices. Percent correct **(A)** and response times (**B**;ms) are represented as a function of the level of averageness (i.e., number of utterances per voice average). Gray dots represent each participant's data point. The black square represents the average performance across listeners. In **(A)**, the dotted line indicates chance level. Black lines: linear regression built using the average slope and intercept values obtained after performing the linear regression in each and every subject. For famous voices, performance increased and RTs decreased significantly with increasing number of utterances per average.

Accuracy and response times, recorded from voice onset, were collected for each participant. Statistical analyses were the same as in experiment 1, but with 5 averageness levels (Table 2; Figure 2). Threshold *T*-values were extracted by taking bootstrap values corresponding to the 95% confidence interval of the sorted bootstrapped T values (accuracy threshold [−1.96 2.29], RTs threshold [−1.92 2.41]).

### Results and discussion

Speaker recognition was above chance level (33%) in all conditions [all Ts_(12)_ > 5.2; all ps < 0.001–Table 2]. A linear regression analysis revealed a significant positive linear relationship between the level of averageness and percent of correct identification {average slope ± s.e.m. [95% CI] = 4.45 ± 0.89 [2.74 6.11]; *T*_(12)_ = 4.997; *p* = 0.0006; Cohen's dz = 1.39–Figure 2A}: participants' accuracy improved with increasing number of vowels per average. Conversely, response times decreased linearly with increasing number of vowels per prototype {average slope ± s.e.m. [95% CI] = −29.14 ± 6.58 [−42.27 −17.43]; *T*_(12)_ = −4.43, *p* = 0.0017; Cohen's dz = 1.23–Figure 2B}. Speaker identification for famous persons was both more accurate and faster for artificially created speaker averages than for the original voices actually uttered by the speakers: the more vowels were averaged the better was recognition.

## Comparison of experiment 1 and 2

To compare regression coefficients across experiments, we performed paired-samples *t*-tests. Significant differences between experiments 1 and 2 on accuracy {*T*_(12)_ = −6.83; *p* < 0.001; threshold T: [−2.11 2.34]} and RTs {*T*_(12)_ = 3.37; *p* = 0.086; threshold T: [−2.39 2.02]} were found. The slope for accuracy was positive in experiment 2 while it was negative in experiment 1, showing that while performance improved with level of averageness for familiar faces, it worsened for trained to familiar voices. For RTs, the slope was significantly larger for familiar voices than trained-to-familiar voices reflecting a significant decrease of RTs with level of averageness for familiar voice recognition only.

In order to test whether differences between experiment 1 and 2 could be attributed to (1) differences in recognition threshold and (2) acoustic characteristics between the famous and trained-to-familiar voices, we performed further analysis. First, we compared recognition accuracy on the original vowel sounds across experiment 1 and 2; this comparison showed no significant difference in the recognition of trained-to-familiar and famous voices [*T*_(12)_ = 1.28; *p* = 0.23]. Then, we analyzed three acoustical parameters (F0, formant dispersion, HNR) of the original voices used in the experiments with Praats (Boersma and Weenink, 2017). Voices are encoded in a multidimensional voice space, which is an acoustic-based representation of voices whose dimensions can be approximated by the fundamental frequency (F0), formant dispersion (i.e., average distance between formants – FD), and the harmonic-noise-ratio (HNR; Latinus et al., 2013); thus, we measured the distances between all the original tokens in that space, in order to ensure that the physical distance between stimuli did not account for the results. For each original vowel we measured the three acoustical parameters, which were then z-scored with respect to the average F0, formant dispersion and HNR across all tokens produced by speakers of the same sex. The Euclidean distance between vowels was then measured; a two sample *t*-test on the Euclidean distance within the voice space showed no differences in physical distance between the trained-to-familiar voices and the famous voices [*T*_(256)_ = −0.094; *p* = 0.92].

## General discussion

Understanding how the human brain recognizes familiar voices is of crucial interest for psychological, and forensic sciences but also for the development of automatic speaker recognition technology. A challenge for these domains outside of psychology is in developing short utterance speaker recognition systems in order to facilitate speaker recognition when only small amounts of data are available (Fatima and Fang Zheng, 2012). Here, we investigated the cognitive mechanisms that enable efficient speaker recognition from very short voice samples. In two experiments, we tested the hypothesis that human listeners rely on speaker averages, built through experience with multiple utterances of a speaker, to recognize familiar speakers. As previous studies have highlighted differences in laboratory-learned and famous voices (Blank et al., 2014), this hypothesis was investigated for both trained-to-familiar voices (Experiment 1) and for voices from public personalities (Experiment 2). We found an advantage of speaker vocal averages over original tokens for the recognition of famous, but not trained-to-familiar, voices.

Our results confirmed that listeners are fairly good at perceiving identity from very short voice sample (less than 500 ms; Van Lancker et al., 1985). Although voice recognition performance is in general, well above chance, voice identification is not infallible (Sherrin, 2015) and performance can appear relatively poor. A large variability in identification performance has been reported across studies, speakers and listeners (Papcun et al., 1989; Lavner et al., 2001; Skuk and Schweinberger, 2013; Sherrin, 2015). In particular, listeners' performance strongly depends on sample duration with longer samples yielding higher accuracy (Schweinberger et al., 1997a; Kreiman and Sidtis, 2011; Skuk and Schweinberger, 2013), with a peak of performance observed for durations between 500 and 1,000 ms (Van Lancker et al., 1985; Schweinberger et al., 1997a). Nevertheless, here participants consistently performed above chance level, even for the speaker averages. Recognition performance on the original vowels was similar across the famous and trained-to-familiar voices, suggesting that the training procedure was sufficient to reach recognition levels similar to real-world familiarized voices. Yet, results of the linear regression analysis suggest a qualitative difference in the recognition of famous and trained-to-familiar voices. For learned voices, performance *decreased* with increasing numbers of voice samples in the averages, suggesting that, for these voices, participants had not extracted/created an abstract representation of the speakers. However, famous voice recognition improved with increasing level of averageness. Even though participants had never heard the averages, their accuracy increased and their reaction times decreased as a function of level of averageness. Andics et al. (2007) previously showed that participants' performance in a voice discrimination task varied with the phoneme used to assess recognition. Better performance was consistently observed for the neutral vowel (the schwa – [ə]) — a relaxed vowel pronunciation produced when the vocal tract is in its neutral state (Andics et al., 2007). In the current study, speaker averages, built by averaging several vowel sounds, resemble the schwa (see Supplementary Material). The schwa is produced by the vocal tract in its neutral state, thus, representative of a speaker's unique vocal tract anatomy, regardless of noise, and environmental factors. The smoothing of intraspeaker variability, naturally occurring due to environmental noise and emotional expressions, during voice averaging achieves the same result (e.g., a neutral, noise-free, voice). This smoothing amplifies the diagnostic information of an individual and results in more stable and robust person representation (Burton et al., 2011; Jenkins and Burton, 2011). A similar advantage of average was described for face recognition: performance of human observers and automatic face recognition improved with face prototypes (Jenkins and Burton, 2008, 2011). The current study demonstrates for the first time the importance of averages in recognizing familiar individuals from their voice.

These studies shed light on the mechanisms involved in the recognition of familiar voices. While previous studies have assumed that voice recognition relies on either a single acoustic feature (e.g., F0) or a universal set of acoustic features (e.g. F0 and first formant; Baumann and Belin, 2010), multiple studies have failed in finding this common set (Lavner et al., 2000; Belin et al., 2011; Kreiman and Sidtis, 2011). This led researchers to suggest that neither a single parameter (Kuwabara and Sagisak, 1995) nor a universal set of parameters (Kreiman and Sidtis, 2011) can account for the perception of voice identity; reasoning instead that voices are recognized as Gestalt-like patterns whereby all acoustical features of an individual voice are processed as a whole and integrated in a voice configuration (Kreiman and Sidtis, 2011; Latinus and Belin, 2012). Results of the studies described here suggest that these patterns can be well approximated by the average of multiple utterances produced by a single speaker. Recognition of famous voices relies on matching incoming vocal sounds to stored representations; we propose that this stored representation, well approximated by the average of multiple utterances of a speaker, corresponds to the individual prototype of a speaker. Thus, familiar voices appear to be encoded as one prototype rather than as a multiple of exemplars under different environmental conditions.

We failed to show a similar effect with the trained-to-familiar voices. This is surprising as participants' performance in the recognition of the trained voices was similar to that observed for famous voice recognition. Moreover, previous studies using a similar learning procedure have demonstrated that the procedure enabled an abstract representation of a speaker's identity, relatively independent from low-level acoustical information, of the speaker's identity (Latinus and Belin, 2011, 2012; Latinus et al., 2011). Despite this, in the current study, trained-to-familiar voice recognition did not improve on individual averages, suggesting that the learning procedure was not sufficient to emulate long-term memory storage of the speaker prototype. Participants were better at recognizing identity from tokens they had heard previously suggesting a more exemplar-based representation of trained-to-familiar voices. Differences between learned and famous voices suggest that in order to become expert in recognizing an individual from his voice, and to be able to generalize across various utterances, one needs to build a robust representation of a vocal identity—an individual norm. It could be hypothesized that with the right amount of exposure to an unfamiliar voice and social motivation, these features may be integrated into a whole and stored in long-term memory as a prototype. The current study does not test this hypothesis, however, it demonstrated that voice learning as it was performed here, despite extensive training and exposure to a large variety of items produced by the speakers, was not enough to enable the construction of stable, norm-based, representations of the speakers.

Questions remain as to why voice learning, despite being efficient in allowing a recognition abstracted from low-level acoustical information, was inefficient in mimicking real-life voice learning, and in creating a prototype: was the duration too short? Or was it a lack of social motivation or interest (Kreiman and Sidtis, 2011)? Indeed one caveat of using voice-learning procedures is that the to-be-learned voices present no personal relevance to the listeners and are not associated to any semantic information. Voice learning has been shown to improve by the concomitant presentation of a face (Sheffert and Olson, 2004; Von Kriegstein et al., 2008). It could be asked whether a voice learning procedure in which the listener is provided with more semantic information about the speaker, thereby raising the social motivation for learning the voice would lead to the emergence of norm-based representation of speaker and to the modulation of the voice space. Possibly, varying the items used in the learning procedure between training sessions would enable the emergence of the prototype, by increasing the demands for a robust representation generalizable across varying items.

## Conclusion

Every speaker produces a signature vocal pattern, in which acoustical features have a specific configuration. Listeners naturally derive important social information from this vocal signature. In the studies described here, we have shown that this signature is well approximated by the average of multiple utterances produced by one speaker. The average voice retains the voice configuration of the speakers, and listeners used that abstract representation to perform familiar voice recognition. Furthermore, we showed that speaker representation based on individual prototypes is present only for ecologically learned identities.

## Author contributions

MF: Ran the experiments, analyzed the data, wrote the manuscript. SL: Helped with data analysis, and writing the manuscript. ML: Conceived and designed the experiments, analyzed the data, wrote the manuscript. All authors have read and approve the final version of the manuscript.

### Conflict of interest statement

The authors declare that the research was conducted in the absence of any commercial or financial relationships that could be construed as a potential conflict of interest.
